# Detection of *Streptococcus mutans* Genomic DNA in Human DNA Samples Extracted from Saliva and Blood

**DOI:** 10.5402/2011/543561

**Published:** 2011-04-10

**Authors:** Alexandre R. Vieira, Kathleen B. Deeley, Nicholas F. Callahan, Jacqueline B. Noel, Ida Anjomshoaa, Wendy M. Carricato, Louise P. Schulhof, Rebecca S. DeSensi, Pooja Gandhi, Judith M. Resick, Carla A. Brandon, Christopher Rozhon, Asli Patir, Mine Yildirim, Fernando A. Poletta, Juan C. Mereb, Ariadne Letra, Renato Menezes, Steven Wendell, Jorge S. Lopez-Camelo, Eduardo E. Castilla, Iêda M. Orioli, Figen Seymen, Robert J. Weyant, Richard Crout, Daniel W. McNeil, Adriana Modesto, Mary L. Marazita

**Affiliations:** ^1^Department of Oral Biology, School of Dental Medicine, University of Pittsburgh, Pittsburgh, PA, USA; ^2^Department of Pediatric Dentistry, School of Dental Medicine, University of Pittsburgh, Pittsburgh, PA, USA; ^3^Center for Craniofacial and Dental Genetics, School of Dental Medicine, University of Pittsburgh, Pittsburgh, PA, USA; ^4^Department of Human Genetics, Graduate School of Public Health, University of Pittsburgh, Pittsburgh, PA, USA; ^5^Clinical and Translational Science Institute, University of Pittsburgh, Pittsburgh, PA, USA; ^6^Dental Public Health/Information Management, University of Pittsburgh, Pittsburgh, PA, USA; ^7^Department of Pedodontics, Istanbul Medipol University, Istanbul, Turkey; ^8^Department of Pedodontics, Istanbul University, Istanbul, Turkey; ^9^ECLAMC, Center for Medical Education and Clinical Research(CEMIC), Buenos Aires, Argentina; ^10^ECLAMC, National Institute of Population Medical Genetics(INAGEMP-CNPq) CEMIC, Buenos Aires, Argentina; ^11^ECLAMC, Hospital de Area El Bolsón, Río Negro, Argentina; ^12^ECLAMC, I Multidisciplinary Institute of Cellular Biology (MBICE), La Plata, Argentina; ^13^ECLAMC, National Institute of Population Medical Genetics (INAGEMP-CNPq) Department of Genetics, Oswaldo Cruz Foundation, Rio de Janeiro, RJ, Brazil; ^14^Department of Genetics, Institute of Biology, Center of Health Sciences, Federal University of Rio de Janeiro, Rio de Janeiro, Brazil; ^15^Department of Periodontics, School of Dentistry, West Virginia University, Morgantown, WV, USA; ^16^Department of Dental Practice and Rural Health, West Virginia University, Morgantown, WV, USA; ^17^Department of Psychiatry, School of Medicine, University of Pittsburgh, Pittsburgh, PA, USA

## Abstract

Caries is a multifactorial disease, and studies aiming to unravel the factors modulating its etiology must consider all known predisposing factors. One major factor is bacterial colonization, and *Streptococcus mutans* is the main microorganism associated with the initiation of the disease. In our studies, we have access to DNA samples extracted from human saliva and blood. In this report, we tested a real-time PCR assay developed to detect copies of genomic DNA from *Streptococcus mutans* in 1,424 DNA samples from humans. Our results suggest that we can determine the presence of genomic DNA copies of *Streptococcus mutans* in both DNA samples from caries-free and caries-affected individuals. However, we were not able to detect the presence of genomic DNA copies of *Streptococcus mutans* in any DNA samples extracted from peripheral blood, which suggests the assay may not be sensitive enough for this goal. Values of the threshold cycle of the real-time PCR reaction correlate with higher levels of caries experience in children, but this correlation could not be detected for adults.

## 1. Introduction

Caries remains the most prevalent noncontagious infectious disease in the world [1] and genetics susceptibility to the disease has become the focus of some research groups that aim to provide new strategies for addressing the problem. Field studies have been immensely facilitated by the development of approaches that allow DNA to be obtained from saliva samples [2]. These samples can then be kept at room temperature for several days to months until extractions can happen in the laboratory. One of the challenges of this line of work is that genetics susceptibility to caries can be masked by the environmental influences to this disease, such as microbial infection, types of diet, and exposure to fluorides.

In regards to microbial colonization, *Streptococcus mutans* is the main species involved with the initiation of the disease [reviewed by [3]]. Consequently, one possibility is that individual susceptibility to colonization by *Streptococcus mutans* will impact future caries experience. However, the traditional quantification of *Streptococcus mutans* with mitis-salivarius-bacitracin agar medium [4] is laborious and requires direct cultivation of plaque samples. A PCR-based method targeting *gtf* (glucosyltransferase) genes of *Streptococcus mutans* was developed as an alternative way to quantify the bacterial infection in humans [5] that is useful as a practical diagnosis system of *Streptococcus mutans* infection.

This work had two main objectives. Since we have access to human DNA samples from saliva, in theory these samples also contain genomic copies of the oral microbial colonization of subjects at the day of sample collection. Therefore, we used the PCR-based method for detecting *gtf* genes of *Streptococcus mutans* as the way to define if subjects were colonized by *Streptococcus mutans* and to test how this data correlates with caries experience. Theinformation could subsequently aidour future genetics studies and allow the inclusion of *Streptococcus mutans* colonization as a covariate in the analysis. The second goal involved detecting genomic DNA copies of *Streptococcus mutans* in human DNA samples extracted from blood. Since *Streptococcus mutans* can be detected in heart valve and atheromatous plaque samples [6, 7], we hypothesize it also can be detected in the circulation. Westudied human DNA samples extracted from peripheral blood to test if genomic DNA copies of *Streptococcus mutans* can be detected by the PCR-based method developed by Yano et al. [5].

## 2. Material and Methods

### 2.1. Human DNA Samples

All samples evaluated in this study were obtained after approval by the Institutional Review Boards from the University of Pittsburgh, Istanbul University, CEMIC (Argentina), and CONEP (Brazil) and written informed consent was obtained from each subject. A total of 1,424 DNA samples were included in this study and they were available from a number of datasets.

(1) From the University of Pittsburgh School of Dental Medicine Dental Registry and DNA Repository (DRDR), 666 DNA samples extracted from whole saliva were utilized. These samples are from consented individuals from Pittsburgh and surrounding regions with ages ranging from 4 to 89 years (average 42.9 years). Individuals that are part of the registry match the demographic distribution of the city of Pittsburgh (approximately 70% White, 20% African American, and the remaining other groups). Approximately 60% of the subjects are females. Most of the individuals are from lower socioeconomic stratum, based on their insurability.

(2) From the Center for Oral Health Disparities in Appalachia (COHRA), 174 DNA samples extracted from whole blood and 44 from saliva were utilized. These samples were from consented families recruited in several sites of rural Pennsylvania and West Virginia. The DNA samples from whole blood were from individuals with ages ranging from 1 to 68 years (average 23.2 years), and the DNA samples from saliva were from children, ages ranging from 1 to 11 years (average 3.3 years).This study population includes a range of socio-economic status (median annual household income less than $25,000), and is fairly representative of the general Appalachian population, which ranks very low compared with the rest of the nation in terms of many oral health measures and access to oral health care. All individuals included in this analysis were White with ratio male:female almost 1.0.

(3) From Istanbul, 175 DNA samples extracted from whole saliva were utilized. These samples are from children whose parents' consented participation in the study. These children were 5 to 6 years of age and 85 were caries-free. These individuals were recruited in the Pedodontics Clinics of Istanbul University and daycare facilities in the city of Istanbul. Seventy-nine were males and 94 females.

(4) From Pittsburgh, 158 DNA samples extracted from whole saliva that did not overlap with the samples from the Dental Registry and DNA Repository were also utilized. These samples were from consented individuals evaluated as a requirement for the University of Pittsburgh School of Dental Medicine students taking the Cariologycourse. These individuals have ages ranging from 13 to 82 years (average 43.3 years) and their characteristics are very similar to the ones described above for the DRDR sampling.

(5) From Guatemala, 109 DNA samples extracted from whole saliva were utilized. These samples are from consented families with children born with oral clefts and controls who received care during a medical mission sponsored by the nonprofit organization Children of the Americas. These samples are from individuals with ages ranging from 14 to 60 years (average 28 years). Forty-five were males and 64 females. Samples from individuals born with clefts were not included in the present study since there is a suggestion these individuals may have a higher incidence of caries.

(6) From Argentina, 98 DNA samples extracted from whole saliva were utilized. These samples are from consented families with children born with oral clefts living in the Patagonian region, from individuals with ages ranging from 10 to 72 years (average 31.5 years). Samples from individuals born with clefts were not included since there is a suggestion that these individuals may have a higher incidence of caries. This study population is basically the admixture of Amerindian Mapuches and Spaniards and come from a lower socioeconomic stratum. The ratio male:female is almost 1.0.

DMFT/dmft (Decayed, Missing due to caries, Filled Teeth) scores were available for all DNA samples utilized in this study and were obtained as recommended by the World Health Organization [1]. They were collected according to international standards. A higher ratio of active carious lesions was seen in younger subjects. For the 174 DNA samples extracted from whole blood, semiquantitative values of *Streptococcus mutans* were also available (assessed from saliva of the same subjects with Dentocult SM Strip mutans, Orion Diagnostica Oy, P.O.Box 83, Espoo, Finland). All 1 424 DNA samples were successfully tested before in other PCR-based genotyping approaches.

### 2.2. DNA Extraction

DNA was extracted from saliva samples using the manufacturer's protocol for manual purification of DNA from 4.0 mL, PD-PR-015 Issue 2.0. Saliva samples had been previously stored at room temperature for up to 3 months in the Oragene vials until DNA extraction. According to the manufacturer of the vials, saliva DNA is stable for over 2 years at room temperature [8]. The entire saliva sample was extracted with reagent volumes adjusted to maximize the amount of DNA recovered. Briefly, samples were mixed by inversion, and then incubated overnight at 50°C. Samples were transferred to a centrifuge tube and mixed with Oragene purifier, incubated on ice, then centrifuged at 3000 × g for 20 minutes to pellet the denatured protein. The supernatant was transferred to a new tube and DNA was precipitated by adding an equal volume of 100% ethanol. The DNA pellet was washed with 70% ethanol, dried, and resuspended with TE buffer. DNA was incubated at 50°C for 1 hour, followed by incubation at room temperature overnight to ensure complete rehydration. A high-speed centrifugation step at 15,000 × g was performed to remove additional impurities. 

DNA was extracted from blood samples using QIAamp DNA Mini Kit according to the manufacturer's instructions.

### 2.3. Real-Time PCR Assay

The assay was based on the experiment developed by Yano et al. [5]. In that work, DNA was extracted from whole saliva from human subjects. Yano et al. [5] validated this method using the standard culture approach as a parameter. They reported that these assay results in regards of measuring *Streptococcus mutans* are strongly correlated with the results obtained from culture. Furthermore, the sensitivity of both methods is very similar, with the real-time PCR able to detect the presence of about 800 copies of genomic DNA from *Streptococcus mutans*. The assay however does not discriminate the specific *Streptococcus mutans* serotypes c, e, or f. Primers used in the assay anneal to conserved regions of *gtf*B and *gtf*C genes of *Streptococcus mutans*, and amplify 415 base pair DNA fragments from both genes. 

Real-time PCR was performed by the use of the ABI PRISM 7900HT Fast Real-Time PCR System (Applied Biosystems). Each reaction tube contained 10 *μ*L of reaction mixture, including 1 X SYBR Green PCR buffer, 0.025 U/uL of AmpliTaqGold DNA polymerase, 0.01 U/uL of AmpErase UNG (uracil N-glycosylase), 1.2 mM of each of the dNTPs, 3 mM MgCL2 (SYBR Green PCR Core Reagents, Applied Biosystems), 2 *μ*L of human DNA extracted from saliva or blood samples and 0.8 mM of each primer specific to *Streptococcus mutans* (forward 5′AGCCATGCGCAATCAACAGGTT3′ and reverse 5′CGCAACGCGAACATCTTGATCAG3′). The specificity of these primers was tested previously [5] and *Streptococcus mutans* genomic DNA (*Streptococcus mutans* ATCC 25175) was included in all reactions as positive control and standard DNA curves were generated. The cycling conditions were 2 minutes at 50°C for uracil *N-glycosylase* (this treatment prevents carryover cross-contamination by digesting uracil-containing fragments generated prior the PCR assay), 10 minutes at 95°C for activation of AmpliTaqGold, 40 cycles of 15 seconds at 95°C for denaturation and 1 minute at 68°C for annealing and extension. Values of the threshold cycle (Ct) above zero were considered positive for *Streptococcus mutans* infection based on the successful amplification of the target sequence. These values were correlated with DMFT scores and alpha of 0.05 was considered statistically significant.

## 3. Results

Our results were compatible with expected values obtained from the validation procedures described by Yano et al. [5]. A subset of samples were tested multiple times at different time points and results agreed. *Streptococcus mutans* genomic DNA was detected in samples from human saliva, both in caries-free individuals and in individuals with previous/current caries experience (Table 1). However, genomic DNA copies from *Streptococus mutans* could not be detected in DNA samples from human peripheral blood (Table 2). These samples were obtained from both adults and children with various levels of caries experience (Table 3). When DMFT/dmft scores were correlated to the values of the threshold cycles of the real-time PCR experiment, statistically significant correlations were only found in the groups comprised exclusively of samples from children (Table 2). Stratifying the data analysis of the adult samples by age did not substantially change the results.

## 4. Discussion

Any study related to the etiology of caries needs to consider the multifactorial nature of the disease. Microbial colonization is a major factor that modulates the initiation of the disease and always needs to be considered. In the case of our studies, in which we have DNA samples extracted from human saliva, the real-time PCR assay developed by Yano et al. [5] to identify the presence of genomic DNA copies of *Streptococcus mutans* in the sample may be utilized as an indication of the presence of its colonization.

We detected the presence of *Streptococcus mutans* genomic DNA copies in a number of caries-free individuals (Table 1). This result may be interpreted in a number of ways. *Streptococcus mutans* in the saliva is necessary but not sufficient to determine the disease and/or the presence of carious lesions. The microorganism may be present in caries free individuals, but their detectable levels are lower. Another possibility is that up to 10% of caries lesions may be missed by the use of the methodology recommended by the World Health Organization without complementation of radiographs [9]. 

Slayton et al. [10] showed that *Streptococcus mutans* levels were positively associated with caries experience in children 3 to 5 years of age. The authors measured levels of *Streptococcus mutans* by microbiological assays, according to the protocol described by Edelstein and Tinanoff [11], by touching a wooden tongue blade to the dorsal surface of the tongue until wet and then using the tongue blade to inoculate selective growth medium for *Streptococcus mutans* (CRT Bacteria Kit, IvoclarVivadent, Schaan, Liechtenstein). Also, a significant interaction was suggested between genetic variation in *tuftelin*, a gene suggested to be involved in enamel formation, and levels of *Streptococcus mutans*. Previously we were not able to replicate the results of a possible interaction between variation in *tuftelin* and levels of *Streptococcus mutans* [12]. We used the same real-time PCR assay described here to define presence or absence of *Streptococcus mutans* colonization in the study. However, our current analysis confirms *Streptococcus mutans* levels are associated with caries experience in children, corroborating the findings by Slayton et al. [10]. Our results clearly show that DMFT scores of adults do not correlate with *Streptococcus mutans* colonization measured by real-time PCR assays. The likely reason is that *Streptococcus mutans* are associated with the initiation of the disease, which usually affects children, and caries experience scores (dmft) are mostly related to the presence of decayed teeth. In adults, DMFT scores are also influenced by the number of teeth missing and filled due to caries and although the DMFT score tends to increase overtime in all individuals, levels of *Streptococcus mutans* will be elevated the most in instances where new carious lesions in enamel are developing. *Streptococcus mutans* counts also correlate with sugar intake and one can argue that individual variation in diet modifies expected bacteria counts, although this hypothesis cannot be easily tested in a cross-sectional study design. These clearly shows the need for a deep understanding of the etiopathogenesis of caries when proposing to study genetic susceptibility to the disease. Our study confirms that in children, levels of *Streptococcus mutans* clearly correlate with higher caries experience and higher susceptibility to the disease.

There is increasing interest in understanding the potential influence of oral health on systemic health. It has been suggested that higher caries rates can be found in individuals with specific systemic diseases such as cardiovascular diseases [13], asthma, and epilepsy [14]. We were not able to detect any levels of *Streptococcus mutans* in the DNA samples extracted from peripheral blood. This may be due to the method used for DNA extraction, which may poorly recover *Streptococcus mutans* genomic DNA in the sample or because of the low level of *Streptococcus mutans* in the original specimen. Evidence exists that *Streptococcus mutans* can be detected in heart valve and atheromatous plaque samples [6, 7] and therefore travel from the mouth to the heart. The explanation is likely that very small numbers of bacteria copies, below the sensitivity of the real-time PCR assay, can be found in any given volume of blood of someone affected. While the concentration of *Streptococcus mutans* varies based on the nature of the sample collected (blood or saliva) and on how it is collected (whole saliva or buffered saliva), DNA isolation methods do not appear to importantly influence the recovery of genomic DNA of *Streptococcus mutans* or the performance of this PCR-based assay [5]. There is great interest in approaches that can help predict how oral health can affect systemic health and future work in our laboratory will aim to identify variables that can serve as predictors of poor systemic health.

Our data provides a rationale for focusing future genetic studies of caries in younger populations, in which levels of *Streptococcus mutans* better correlate with existing measures of disease experience, and sampling tends to be more homogeneous.

## Figures and Tables

**Table 1:** Frequency of *Streptococcus mutans* colonization in the population studied.

Cohort		*N*	Positive for *S. mutans* colonization
Argentina	Caries-free	24	10
	Caries-affected	74	48

Guatemala	Caries-free	26	9
	Caries Affected	83	39

Turkey	Caries Free	85	18
	Caries Affected	90	68

Dental registry and DNA repository (DRDR)	Caries Free	28	24
	Caries Affected	638	471

Cariology course	Caries Free	2	1
	Caries Affected	156	123

Center for oral health disparities in appalachia (COHRA) DNA from saliva	Caries Free	21	11
	Caries Affected	23	20

Center for oral health disparities in appalachia (COHRA) DNA from blood	Caries Free	44	12*
	Caries Affected	130	114*

*As determined by the use of Dentocult SM Strip mutans (North Bay/BioScience, LCC; Traverse City, MI, USA) assessed from saliva of the same subjects.

**Table 2:** Results of Pearson's correlation test between caries experience and values of the threshold cycle of the real time PCR experiment.

Population	*N*	DMFT/dmft average	rs statistics	*T* statistics	Degrees of freedom*	Two-tailed *P*-values
Argentina	98	7.44	−0.0831	−0.82	96	.41
Guatemala	109	6.87	0.07244	0.7513	107	.45
Turkey	175	3.68	0.3328	3.23	84	.002
Dental Registry and DNA Repository (DRDR)	666	15.98	0.02069	0.53	655	.6
Cariology Course	158	3.77	0.05079	−0.6352	156	.53
Center for Oral Health Disparities in Appalachia (COHRA) DNA from saliva	44	1.64	0.4343	3.161	43	.003
Center for Oral Health Disparities in Appalachia (COHRA) DNA from blood^ #^	174	6.91	No *Streptococcus mutans* detected			
Total	1424					

*Differences between *N* and degrees of freedom bigger than 1 are due to samples that failed the assay. The control caries-free group from Turkey was not included in this analysis. ^#^Distribution of the samples according to Dentocult TM Strip mutans scores assessed from saliva od the same subjects: 44 (<1 × 10^5^ CFU/mL); 45 (<1 × 10^5^ CFU/mL); 44 (1 × 10^5^ – 1 × 10^6^ CFU/mL); 41 (>1 × 10^6^ CFU/mL).

**Table 3:** Caries experience scores of the studied population.

Population	*N*	Average DMFT/dmft	Average age (years)
Argentina	98	7.44	31.5
Guatemala	109	6.87	28
Turkey	175	3.68	5.4
DRDR	666	15.98	42.9
Cariology Course	158	3.77	43.3
COHRA saliva	44	1.64	3.3
COHRA blood	174	6.91	23.2
